# Repeat-Associated Non-AUG Translation of AGAGGG Repeats that Cause X-Linked Dystonia-Parkinsonism

**DOI:** 10.1002/mds.29183

**Published:** 2022-08-16

**Authors:** Charles Jourdan Reyes, Katsura Asano, Peter K. Todd, Christine Klein, Aleksandar Rakovic

**Affiliations:** 1 Institute of Neurogenetics, University of Lübeck, Lübeck; 2 Molecular Cellular and Developmental Biology Program, Division of Biology, Kansas State University, Manhattan, Kansas, USA; 3 Laboratory of Translational Control Study, Graduate School of Integrated Sciences for Life, Hiroshima University, Hiroshima, Japan; 4 Hiroshima Research Center for Healthy Aging, Hiroshima University, Hiroshima, Japan; 5 Department of Neurology, University of Michigan Medical School, Ann Arbor, Michigan, USA; 6 Veterans Affairs Medical Center, Ann Arbor, Michigan, USA

**Keywords:** X-linked dystonia-parkinsonism, RAN translation, repeat expansion, SVA retrotransposon, Dipeptide repeat proteins, TAF1

## Abstract

**Background:**

X-linked dystonia-parkinsonism (XDP) is a neurodegenerative disorder caused by the intronic insertion of a SINE-VNTR-*Alu* (SVA) retrotransposon carrying an (AGAGGG)_*n*_ repeat expansion in the *TAF1* gene. The molecular mechanisms by which this mutation causes neurodegeneration remain elusive.

**Objectives:**

We investigated whether (AGAGGG)_*n*_ repeats undergo repeat-associated non-AUG (RAN) translation, a pathogenic mechanism common among repeat expansion diseases.

**Methods:**

XDP-specific RAN translation reporter plasmids were generated, transfected in HEK293 cells, and putative dipeptide repeat proteins (DPRs) were detected by Western blotting. Immunocytochemistry was performed in COS-7 cells to determine the subcellular localization of one DPR.

**Results:**

We detected putative DPRs from two reading frames, supporting the translation of poly-(Glu-Gly) and poly-(Arg-Glu) species. XDP RAN translation initiates within the (AGAGGG_*n*_ sequence and poly-(Glu-Gly) DPRs formed nuclear inclusions in transfected cells.

**Conclusions:**

In summary, our work provides the first in-vitro proof of principle that the XDP-linked (AGAGGG)_*n*_ repeat expansions can undergo RAN translation. © 2022 The Authors. *Movement Disorders* published by Wiley Periodicals LLC on behalf of International Parkinson and Movement Disorder Society

DNA tandem repeats are the most unstable portions of the human genome.^1^ Their expansion causes more than 50 genetic diseases,^1^ most of which are neurological.^2^ How these dynamic mutations elicit neurodegeneration are variable dependent on the location of the repeats within genes and their impact on transcription and translation. Historically, non-coding repeat expansions were thought to cause disease exclusively by host gene loss of function or RNA-mediated toxicity.^3^ However, repeats can also support non-canonical translation in the absence of an AUG start codon through a process known as repeat-associated non-AUG (RAN) translation.^4^ RAN translation generates aggregation-prone proteins from multiple reading frames, contributing to the pathogenesis of at least 10 repeat expansion disorders.^3^

X-linked dystonia-parkinsonism (XDP) is a hereditary neurodegenerative disorder with a clear genetic etiology, but ill-defined pathophysiology. XDP involves a progressive neuronal loss in the striatum and neuroanatomic alterations in the cortex and cerebellum.^5–7^ Recent genetic findings converge on the notion that the age-related penetrance and expressivity of XDP are modified by an (AGAGGG)_*n*_hexanucleotide repeat expansion within a SINE-VNTR-*Alu* (SVA) retrotransposon inserted in the sense strand (and [CCCTCT]_*n*_ in the antisense orientation) of the *TAF1* gene.^8,9^ Repeat length ranged from 30 to 55 in patients and showed a significant inverse correlation with age at disease onset (range, 22–67 years).^9^ *TAF1* loss of function (LoF) was proposed as the primary mechanism causing striatal neurodegeneration in XDP.^10,11^ However, *TAF1* LoF in rodents’ brains failed to recapitulate the specific pattern of striatal neurodegeneration in this rare disease.^12,13^ Moreover, somatic expansions of the hexanucleotide repeat mentioned above were found to occur at greater levels in the brain relative to the blood of XDP patients, hinting at the possible contribution of other repeat-mediated pathogenic processes in XDP.^14–16^ This study investigated the potential of the XDP-linked (AGAGGG)_*n*_ repeat expansion to undergo RAN translation as a possible contributor to disease pathogenesis.

## Methods

### Development of XDP-Specific RAN Translation Reporter Plasmids

Detailed procedures for generating XDP-specific RAN translation reporter plasmids are found in the Supporting Data (including Supplementary Table S1).

### Mammalian Cell Culture, Transfection Conditions, and Western Blot Analysis

Human embryonic kidney 293 (HEK293) cells were maintained in Dulbecco’s modified Eagle medium (DMEM) supplemented with 10% fetal bovine serum (FBS) and 1% penicillin/streptomycin (Gibco, Grand Island, NY) at 37°C in the presence of 5% CO_2_. For transfection, cells were seeded in 6-well plates at 2.0 × 10^6^ cells per well and transfected using a mixture of 5 μg of plasmid DNA, 20 μL FuGENE HD transfection reagent (Promega, Madison, WI), and 300 μLof sterile Opti-MEM 24 h post-seeding. Cells were then lysed 24 h after transfection with radioimmunoprecipitation assay (RIPA) buffer (Pierce, Rockford, IL). The protein concentration was determined using the Pierce BCA protein assay kit (Thermo Scientific, Waltham, MA) immediately before Western blot analysis.

For Western blot analysis, 10 μg of protein was prepared in 1x NuPAGE LDS sample buffer (Thermo Scientific) and electrophoresed in 1.0-mm, 10-well NuPAGE 4%–12% Bis-Tris protein gels (Thermo Scientific, Waltham, MA) at 150 V for 90 min. Proteins were transferred to a nitrocellulose membrane at 32 V for 60 min. Visualization was carried out using the SuperSignal West Pico PLUS Chemiluminescent Substrate (Thermo Scientific, Waltham, MA), the ChemiDoc MP Imaging System (Bio-Rad Laboratories, Hercules, CA), and the appropriate antibodies in Supplementary Table S2.

### Immunocytochemical Staining and Apoptosis Assay

COS-7 cells were seeded on coverslips and carefully placed in 24-well plates. After 24 h, each well was transfected using 33 μL of the aforementioned transfection mixture. Twenty-four hours post-transfection, cells were fixed in 4.0% formaldehyde for 15 min and permeabilized with 0.1% Triton X-100. Blocking was performed using 4.0% normal goat serum in phosphate buffered saline (PBS) for 1 h. Immunofluorescence staining was done using a primary antibody against FLAG (1:100; Sigma-Aldrich). The appropriate secondary antibody was used (Goat anti-mouse IgG Alexa Fluor 594, 1:1000, Invitrogen). Coverslips were viewed using an LSM710 confocal microscope (Zeiss, Oberkochen, Germany). Apoptosis was assayed using a TUNEL Apoptosis Assay Kit (Roche Life Science, Basel, Switzerland) according to the manufacturer’s protocol. TUNEL-positive cells were counted in 10 randomly chosen viewing fields each containing at least 20 cells. Coverslips were viewed using an LSM710 confocal microscope (Zeiss, Oberkochen, Germany) under 40× magnification.

## Results

### The (AGAGGG)_*n*_ Sequence Is RAN-Translated in Two Reading Frames

**FIG. 1.**
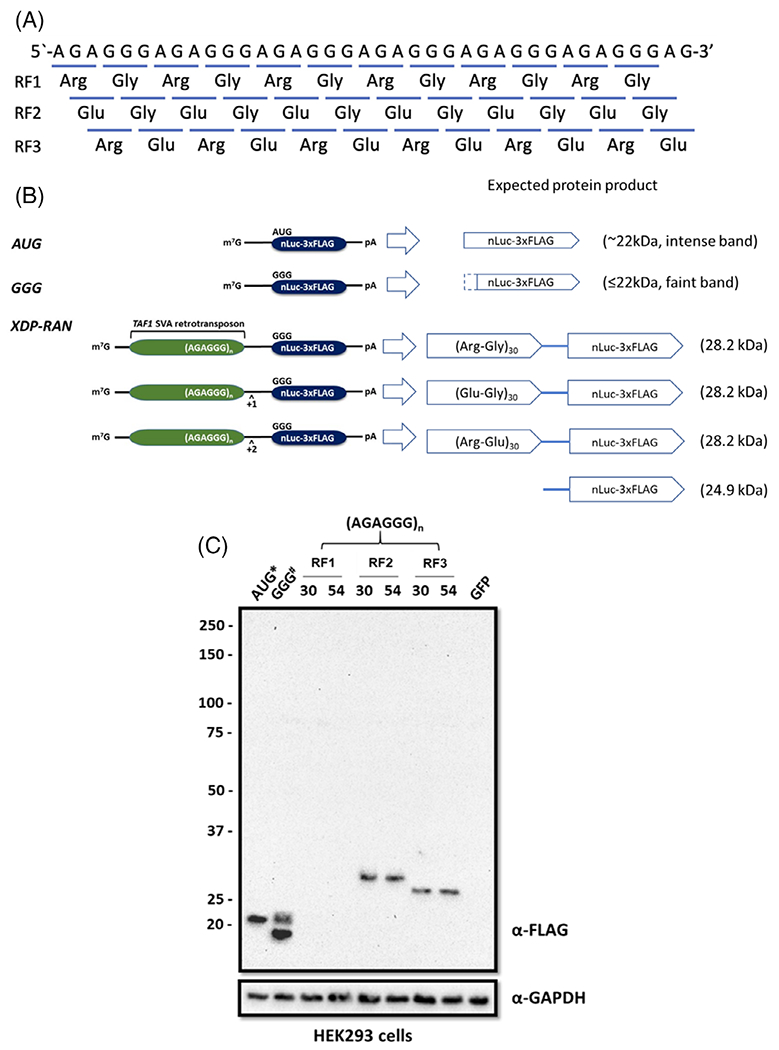
Development of repeat-associated non-AUG (RAN) translation-specific reporter plasmids for X-linked dystonia-parkinsonism (XDP). (**A**) Schematic depiction of the putative dipeptide repeat proteins (DPRs) generated by RAN translation of the (AGAGGG)_*n*_ repeats in the three alternate reading frames (RF1, RF2, and RF3). (**B**) Plasmids were designed by inserting the *TAF1* SVA retrotransposon bearing 30 or 54 AGAGGG repeats upstream of a mutant nanoluciferase (nLuc) with the canonical AUG start site replaced by a GGG codon. The SVA was inserted in 3 different reading frames (RF1, RF2, and RF3) relative to the repeat sequence. A C-terminal 3xFLAG tag is available for Western blot detection. m7G = 7-methylguanosine cap; pA = poly(A) tail. (**C**) Western blot analysis using a FLAG-specific antibody in HEK293 cells transfected with XDP-specific RAN translation reporter plasmids carrying 30 or 54 repeats indicates that the (AGAGGG)_*n*_ sequence is translated in RF2 and RF3, forming putative poly-(Glu-Gly) and poly-(Arg-Glu) DPRs. No band was detected in RF1 (poly-[Arg-Gly]) reading frame. In addition, no apparent molecular weight increase was observed between 30- and 54-repeat constructs. GAPDH was used as a loading control. *One-hundredth of the AUG control plasmid was used to avoid overexposure on western blots. #Smaller molecular weight proteins derived from initiation within the nLuc tag coding sequence were observed in the GGG control. A GFP-expressing vector (pmaxGFP) was used to determine successful transfection.

Based on the hexanucleotide repeat motif located within the XDP-causing intronic SVA retrotransposon, a maximum of three dipeptide repeat proteins (DPRs) could potentially cause neurodegeneration if the XDP-associated repeats undergo RAN translation (Fig. 1A). We designed a series of XDP-specific RAN translation reporter plasmids by inserting the *TAF1* SVA retrotransposon bearing either 30 or 54 repeats, the lowest and one of the highest repeat lengths detected in our cohort of patients,^9^ into a previously described RAN translation reporter (GGG-nLuc-3xFLAG) (Supporting Data).^17,18^ Such constructs harbor the nanoluciferase (nLuc)-coding sequence without the canonical AUG start codon followed by a 3xFLAG sequence, therefore, producing fusion proteins initiated only from the upstream sequence (Fig. 1B). These reporter plasmids have been previously used to elucidate how RAN translation is regulated in the context of the *C9ORF72* and *FMR1* repeat expansions causing amyotrophic lateral sclerosis/frontotemporal dementia (ALS-FTD) and fragile X tremor ataxia syndrome (FXTAS), respectively.^17,18^

Western blot analysis of cell lysates derived from HEK293 cells transfected with the RAN translation reporters (Supplementary Table S2) was performed using a FLAG-specific antibody to investigate whether translation could initiate from within the *TAF1* SVA retrotransposon sequence. We observed that the AUG-nLuc-3xFLAG fusion protein was at its expected molecular weight of ~22 kDa. No band was detected in reading frame 1 (RF1), demonstrating the absence of a poly-(Arg-Gly) product. Conversely, bands representing higher molecular weight products were detected in cells expressing either RF2 or RF3, supporting the translation of poly-(Glu-Gly) and poly-(Arg-Glu) DPRs, respectively (Fig. 1C). In both reading frames, no repeat length-dependent shift in molecular weight and slower migrating proteins accumulating in the stacking gel were observed between 30- and 54-repeat plasmids.

### RAN Translation Initiates within the (AGAGGG)_*n*_ Sequence

**FIG. 2.**
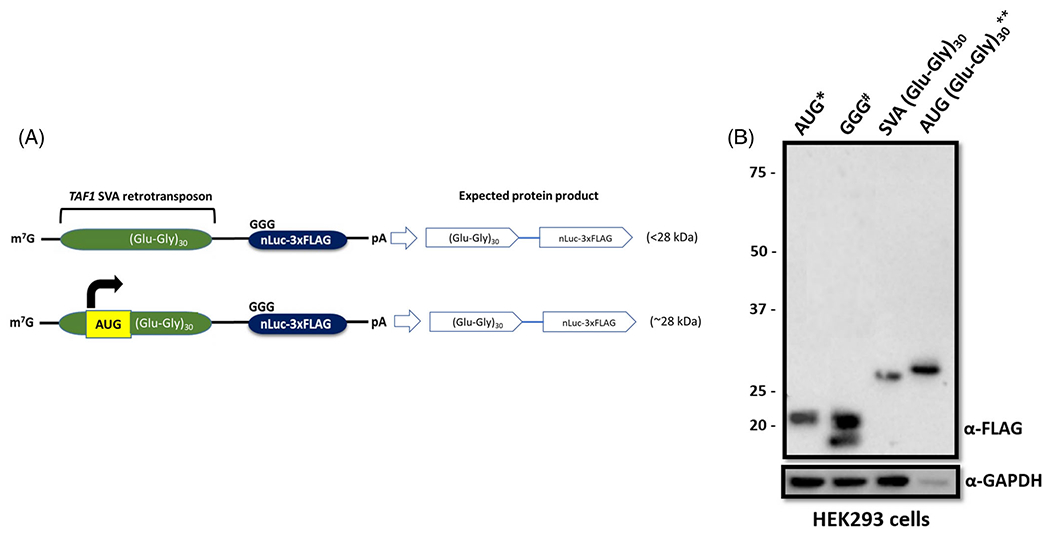
Repeat-associated non-AUG (RAN) translation initiates within the (AGAGGG)_*n*_ repeat sequence. (**A**) Translation initiation was forced upstream of the repeat sequence by artificially introducing an AUG start codon in good Kozak sequence context (yellow box) in the reading frame 2 (RF2). (**B**) Western blot analysis using a FLAG-specific antibody in HEK293 cells transfected with 30-repeat constructs in the native or AUG-initiated context. GAPDH was used as a loading control. *One-hundredth of the AUG control plasmid was used to avoid overexposure on Western blots. #Smaller molecular weight proteins derived from initiation within the nanoluciferase (nLuc) tag coding sequence were observed in the GGG control. **The cell lysate was diluted 10-fold to avoid signal saturation on Western blots.

Depending on the sequence context, RAN translation can initiate from near-cognate start codons upstream of the repeats or within the repeat motif forming a secondary structure such as a hairpin or G-quadruplex.^17^ The molecular weight of the bands (Supplementary Table S3) and the absence of a repeat length-dependent increase in molecular weight between 30- and 54-repeat constructs in both reading frames suggested that RAN translation initiates within the (AGAGGG)_*n*_ sequence. To verify this hypothesis, translation was forced to begin directly upstream of the hexanucleotide repeats by introducing an AUG start codon in a good Kozak sequence context in RF2 (Fig. 2A). HEK293 cells were transfected with 30-repeat plasmids in the native and AUG-initiated sequence contexts. Western blotting using the same FLAG-specific antibody showed that the AUG-initiated plasmid produced a higher molecular weight protein than the original plasmid, supporting the proposed mechanism for translation initiation (Fig. 2B).

### Poly-(Glu-Gly) Has a Propensity to Form Nuclear Inclusions

Immunocytochemical staining was performed in COS-7 cells to determine the subcellular localization of the poly-(Glu-Gly) DPR. In contrast to the AUG-nLuc-3xFLAG fusion protein, which was widely distributed throughout the nucleus and the cytoplasm, poly-(Glu-Gly) was primarily localized in the nucleus, where it showed a tendency to form inclusions (Fig. S1A).However, poly-(Glu-Gly) did not induce apoptosis in our model system (Fig. S1B).

## Discussion

Although two independent genome-editing studies have established that the SVA retrotransposon insertion in *TAF1* causes XDP,^19,20^ the pathogenic mechanisms linking this genetic etiology to neurodegeneration in this rare disease remain unsettled. An essential clue to this longstanding issue was the observation that a polymorphic hexanucleotide repeat within this pathogenic insertion correlates with clinical disease manifestation and somatically expands in patients’ brains,^8,9,14,15^ implying that XDP may share causal pathways with other diseases caused by unstable repeat expansions.^16^ By developing XDP-specific RAN translation reporters, our work provides the first *in-vitro* proof of principle that the XDP-associated hexanucleotide repeat is translated into proteins. Importantly, we offer evidence that RAN translation initiates from within the (AGAGGG)_*n*_ repeat, not the upstream sequence. Consistent with this notion, the *TAF1* SVA retrotransposon possessed intrinsic promoter activity,^8^ and SVA transcription has been confirmed to initiate from within the hexamer.^21,22^ Furthermore, a similar regulatory mechanism was recently reported for a 68-bp repeat expansion in *WDR7*, which is transcribed into microRNAs at regular repeat length intervals.^23^

This study reports the potential translation of poly-(Glu-Gly) and poly-(Arg-Glu) DPRs in XDP. To our knowledge, poly-(Glu-Gly) and poly-(Arg-Glu) DPRs have not been reported for any other repeat expansion disorder. Nevertheless, arginine-rich DPRs were the most toxic proteins produced in *C9ORF72*-mediated ALS-FTD, causing neurodegeneration by blocking global protein translation^24,25^ and disrupting nucleocytoplasmic transport.^26^ In particular, poly-(Gly-Arg) DPRs accumulated in clinically affected brain regions^27^ and caused TDP-43 cytoplasmic mislocalization and aggregation,^28^ the main pathological hallmark of ALS-FTD. Because the toxicity of protein aggregates is linked to their specific pathophysiology,^26,29,30^ functional studies are warranted to elucidate the unique cellular pathways disrupted by these putative DPRs in endogenous disease models of XDP.

Our study also encountered some technical limitations. In line with previous observations on FXTAS, RAN translation of the XDP-specific (AGAGGG)_*n*_ repeats was highly inefficient, showing <1% efficiency relative to canonical AUG translation.^31^ Furthermore, the near-cognate AGG initiation codon, which repeatedly occurs within the (AGAGGG)_*n*_ repeat motif, did not show significantly higher expression levels than the GGG-nLuc-3xFLAG negative control in a prior investigation.^32^ These limitations precluded us from directly detecting the nLuc tag via luciferase assays, immunofluorescence, and immunocytochemistry. RAN translation is a highly inefficient process relative to canonical AUG translation, and the slow accumulation of toxic proteins has been linked to the age-associated penetrance of these diseases.^17,31^ However, cellular stress triggered by protein misfolding can promote RAN translation while suppressing global AUG translation.^18,33^ Furthermore, DPR toxicity can synergize with RNA-mediated toxicity mediated by G-quadruplexes^34^ and host gene loss of function to drive progressive neurodegeneration.^35^ Therefore, the subtle build-up of DPRs in aging neurons over time, in concert with other disease-causing pathways, may lead to late-onset neuronal death seen in patients with XDP.^7^

Collectively, our findings support the notion that RAN translation is possible in the context of the XDP-linked (AGAGGG)_*n*_ repeat expansion. Our work provides the impetus for investigating the pathological significance of proteotoxicity in this devastating neurological disease.

## Supplementary Material

sup material

Additional Supporting Information may be found in the online version of this article at the publisher’s web-site.

## Data Availability

The authors confirm that the data supporting the findings of this study are available within the article and its supplementary material.

## Data Availability Statement

The authors confirm that the data supporting the findings of this study are available within the article and its supplementary material.
