# Supplementary material for: Repeat-Associated Non-AUG Translation of AGAGGG Repeats that Cause X-Linked Dystonia-Parkinsonism
Source: Mov Disord. Author manuscript; Available in PMC 2026 May 22. (PMC13197201; doi:10.1002/mds.29183)
Supplement: sup material [file NIHMS2172834-supplement-sup_material.docx]

**SUPPLEMENTARY MATERIALS**

## **Development of XDP-specific RAN translation reporter plasmids**

In brief, the *TAF1* SVA insertion was PCR-amplified from blood-derived genomic DNA as previously described (Supplementary Table 1), and the resulting amplicon was diluted 100-fold. The diluted amplicon was then used as a template for another PCR reaction using three different reverse primers to clone the SVA element in reading frame 1, 2, and 3 (Supplementary Table 1). The amplicons were then digested with HindIII-HF and NheI-HF (New England Biolabs, MA, USA) at 37°C for 1 hour and resolved in 1.0% agarose gel. Bands corresponding to the SVA were extracted from the gel following the prescribed procedure in the QIAquick® gel extraction kit (Qiagen). Subsequently, 5 μL purified digested product was ligated to 3 μL HindIII-HF- and NheI-HF-digested GGG-nLuc-3xFLAG in a reaction mixture with 1 μL T4 DNA ligase and T4 buffer (New England Biolabs, MA, USA). Ligation was set at 1 hour at room temperature before denaturation at 65°C for 10 minutes. The ligation mixture was used to transform One Shot^TM^ Stbl3^TM^ chemically competent *Escherichia coli* cells (Thermo Scientific). The resulting colonies were screened by first extracting plasmids using the QIAprep® Spin Miniprep Kit (Qiagen) and digesting them with HindIII-HF and NheI-HF (New England Biolabs, MA, USA) for 1 hour at 37°C. The correct reading frames were verified by Sanger sequencing using the SVA EAR F (5’-TCACTTCCTAGATGTGATGG-3’) and nLuc R primer (5’-ACATGGATGTCGATCTTCAGC-3’). AUG-initiated constructs were prepared following the same general procedures except that an AUG start codon in good Kozak sequence context was introduced upstream of the AGAGGG repeat via the SVA ATG F primer (Supplementary Table 1).

**Table S1.** Primers and PCR conditions

| **OBJECTIVE** | **PRIMERS** | **PCR KIT** | **AMPLIFICATION PROFILE** |
| --- | --- | --- | --- |
| ***TAF1* SVA retrotransposon amplification** | **Forward**  5’-GTTCCATTGTGTGGTTGTACCAGCGTTTGTTC-3’  **Reverse**  5’-CACATGAAAAGATGCCCAACATCATTAGCCATTAG-3’ | KOD  (Merck Millipore) | 98°C 2min; 5 x (98°C 10 s, 74°C 3min 30s); 5 x (98°C 10s, 72°C 3min 30s); 5 x (98°C 10s, 70°C 3min 30s); 20 x (98°C 10s, 68°C 3min 30s) |
| **XDP-specific**  **RAN translation reporter plasmids** | **Forward**  5’- GAATAGAATGCTAGCTGTTTAGTTTTACAAGACACGGC -3’  **Reverse**  **ORF1**  5’-CAATGCCAGAAGCTTTTTTTTCCACTTCTGATGTGGAA-3’  **ORF2**  5’-CAATGCCAGAAGCTTTTTTTTTCCACTTCTGATGTGGAA-3’  **ORF3**  5’-CAATGCCAGAAGCTTTTTTTTTTCCACTTCTGATGTGGAA-3’ | KOD  (Merck Millipore) | 98°C 2min; 5 x (98°C 10s, 68°C 3min 30s); 5 x (98°C 10s, 66°C 3min 30s); 5 x (98°C 10s, 64°C 3min 30s); 20 x (98°C 10s, 62°C 3min 30s) |
| **ATG-initiated**  **RAN translation**  **reporter plasmids** | **Forward**  5’- <TCCAGCTTTGGCTAGCCACCATGAGAGGGAGACCG> -3’  **Reverse**  **ORF1**  5’-CAATGCCAGAAGCTTTTTTTTCCACTTCTGATGTGGAA-3’  **ORF2**  5’-CAATGCCAGAAGCTTTTTTTTTCCACTTCTGATGTGGAA-3’  **ORF3**  5’-CAATGCCAGAAGCTTTTTTTTTTCCACTTCTGATGTGGAA-3’ | KOD  (Merck Millipore) | 98 °C 2min; 5 x (98 °C 10s, 64°C 3min 30s); 5 x (98°C 10s, 62°C 3min 30s); 5 x (98°C 10s, 60°C 3min 30s); 20 x (98°C 10s, 58°C 3min 30s) |

**Table S2.** Antibodies

| **ANTIBODY** | **DILUTION** | **MANUFACTURER** |
| --- | --- | --- |
| Monoclonal ANTI-FLAG® M2 | 1:1000 for Western blots  1:100 for immunostaining | Sigma-Aldrich  (F3165) |
| Monoclonal Anti-GAPDH | 1:10000 for Western blots | Cell Signaling |
| Goat Anti-mouse IgG, Alexa 594 | 1:1000 for immunostaining | Invitrogen |

**Table S3.** Putative translation initiation sites and the molecular weights of the resulting proteins

| **PLASMID** | **TRANSLATION INITIATION SITE** | **MOLECULAR WEIGHT**  **(kDa)** |
| --- | --- | --- |
| AUG-nLuc-3xFLAG | AUG codon in  nLuc sequence | 22.0 |
| SVA (AGAGGG)_30_  -nLuc-3xFLAG (RF2) | Sequence between AGAGGG repeat  and nLuc | 24.9 |
| SVA (AGAGGG)_30_  -nLuc-3xFLAG (RF2) | Upstream of (AGAGGG)_30_ | 28.2 |
| SVA (AGAGGG)_54_  -nLuc-3xFLAG (RF2) | Upstream of (AGAGGG)_54_ | 30.9 |

***TAF1* SVA retrotransposon [with (AGAGGG)_n_ ] and vector sequences**

**>SVA**

**TTTTTTTTTTTTTTTTTTTTTTTTTTTTTTATT**

**TTTTTTTTATTTTTTTTTTAATTTATTTTTTTATTGATAATTCTTGGGTGTTTCTCACAG**

**AGGGGGATTTGGCAGGGTCATGGGACAATAGTGGAGGGAAGGTCAGCAGATAAACAAGTG**

**AACAAAGGTCTCTGGTTTTCCTAGGCAGAGGACCCTGCGGCCTTCCGCAGTGTTTGTGTC**

**CCTGATTACTTGAGATTAGGGATTGGTGATGACTCTTAACGAGCATGCTGCCTTCAAGCA**

**TCTGTTTAACAAAGCACATCTTGCACCGCCCTTAATCCATTTAACCCTGAGTGGACACAG**

**CACATGTTTCAGAGAGCACAGGGTTGGGGGTAAGGTCACAGATCAACAGGATCCCAAGAC**

**AGAGGAATTTTTCTTAGTGCAGAACAAAATGAAAAGTCTCCCATGTCTACTTCTTTCTAC**

**ACAGACACGGCAACCATCCGATTTCTCAATCTTTTCCCCGCCTTTCCCGCCTTTCTATTC**

**CACAAGGCCGCCATTGTCATCCTGGCCCGTTCTCAATGAGCTGTTGGGCACACCTCCCAG**

**ACCGGGTGGTGGCTGGGCAGAGGCGCCCCTCACCTCCCGGACAGGGCGGCTGGCCGGGCG**

**GGGGGGGCTGACCCCCCCCACCTCCCTCCCGGACGGGGCGGCTGGCTGGGCAGAGGGGCT**

**CCTCACTTCCCAGTAGGGGCAGCCGGGCAGAGGCGCCCCTCACCTCCCGGACGGGGCCAC**

**TGGCCGGGCAGGGGGGCTGACCCCCCCCACCTCCCTCCCGGACGGGGCGGCTGGCCGGGC**

**GGGGGGCTGACCCCCCCACCTCCCTCCCGGACGAGGCGGCTGGCCGGGCGTGGGGCTGAC**

**ACCCCCACCTCCCTCCCGGACAGGGCGGCTGGCCGGGCGGGGGGCTGACCCCCCCACCTC**

**CCTCCCGGATGGGGCGGCTGGTCGGGCGGGGGGCCGACCCCCCCACCTCCCTCCCGGACG**

**GGGCGGCTGGCCGGGCAGAGGGGCTCCTCACTTCCCAGTAGGGGCGGCCGGGCAGAGGCG**

**CCCCTCACCTCCCAGACGGGGCGGCTGGCCGGGCGGAGGGCTGACCCCCCCACCTCCCTC**

**CCGGACAGGGCGGCTGGCCGGGCGGGGGGCTGACCCCCCCACCTCCCTCCCGGACGGGGC**

**GGCTGGCCTGGCAGAGGGGCTCCTCACTTCCCAGTAGGGGCGGCCGGGCAGAGGCGCCCC**

**TCACCTCCCAGACGGGGCGGCTGGCCGGGCGGGGGGCTGACCCCCCCACCTCCCTCCCGG**

**ACGGGGCGGCTGGCCAGGCGGGGGGCTGACCCCCCCACCTCCCTCCCGGACGGGGCGGCT**

**GGCCGGGTGGGGGGGCTGACCCCCCCATCTCCCTCCCGGACGGGGTGGCTGGCCGGGCTG**

**AGGGGCTCCTCACTTCCCAGTAGGGGCGGCCGGGCAGAGGCGCCCCTCACCTCCCGGACG**

**GGGCGGCTGGCCGGGCGGGGGGCTGACCCCCCCACCTCCCTCCCGGATGGCACGGCTGGC**

**CGGGCGGGGGGGCTGACCCCCCACCTCCCTCCCGGATGGGGCGGCTGGCCGGGTGGGGGG**

**CTGACCCCCCCCCACCTCCCTCCCGGACGGGGTGGCTGCTGGGCGGAGATGCTCCTCACT**

**TCCCAGATGGGGTGGCTGCCGGGCGGAGAGGCTCCTCACTTCTCAGACGGGGCAGCTGCC**

**GGGCGGAGGGGCTCCTCACTTCTCAGACGGGGTGGTTGCCAGGCAGAGGGTCTCCTCACT**

**TCTCAGACGGGGCGGCCGGGCAGAGACGCTCCTCACCTCCCAGACGGGGTCTCGGCCGGG**

**CAGAGGCGCTCCTCACATCCCAGATGGGGCGGCGGGGCAGAGGCGCTCCCCACATCTCAG**

**ACGATGGGCGGCCGGGCAGAGACGCTCCTCACTTCCTAGATGTGATGGCGGCCGGGAAGA**

**GGTGCTCCTCACTTCCTAGATGGGATGGCGGCCGGGCGGAGACGCTCCTCACTTTCCAGA**

**CTGGGCAGCCAGGCAGAGGGGCTCCTCACATCCCAGACGATGGGCGGCCAGGCAGAGACA**

**CTCCTCCCTTCCCAGACGGGGTGGCGGCCGGGCAGAGGCTGCAATCTCGGCACTTTGGGA**

**GGCCAAGGCAGGCGGCTGGGAGGTGTGGGTTGTAGTGAGCCGAGATCACGCCACTGCACT**

**CCAGCCTGGGCACCATTGAGCACTGAGTGAACGAGACTCCGTCTGCAATCCCGGCACCTC**

**GGGAGGCCGAGGTTGGCGGATCACTCGCGGTTAGGGGCTGGAGACCGGCCCGGCCAACAC**

**AGCGAAACCCTGTCTCCACCAAAACCAGTCAGGCGTGGCGGCGCGTGCCTGCAATCGCAG**

**GCACTCGGCAGACTGAGGCAGGAGAATCAGGCAGGGAGGATGCAGTGAGCCGAGATGGCA**

**GCAGTACAGTCCAGCTTTGGCTCCGCATGAGAGGGAGACCGTGGGGAGAGGGAGAGGGAG**

**G AGAGGG AGAGGG AGAGGG AGAGGG AGAGGG AGAGGG AGAGGG AGAGGG AGAGGG AGAGG**

**G AGAGGG AGAGGG AGAGGG AGAGGG AGAGGG AGAGGG AGAGGG AGAGGG AGAGCTATTTC**

**ATTTTTTTTTTTTCCTATTTCATTTTTTTTTTTTCCA**

This is the original *TAF1* SVA sequence discovered by Makino et al. 2007 containing 18 (AGAGGG)_n_ repeats highlighted in yellow.

https://www.ncbi.nlm.nih.gov/pmc/articles/PMC1821114/

**>GGGnLuc3xFLAG**

**GACGGATCGGGAGATCTCCCGATCCCCTATGGTGCACTCTCAGTACAATCTGCTCTGATGCCGCATAGTT**

**AAGCCAGTATCTGCTCCCTGCTTGTGTGTTGGAGGTCGCTGAGTAGTGCGCGAGCAAAATTTAAGCTACA**

**ACAAGGCAAGGCTTGACCGACAATTGCATGAAGAATCTGCTTAGGGTTAGGCGTTTTGCGCTGCTTCGCG**

**ATGTACGGGCCAGATATACGCGTTGACATTGATTATTGACTAGTTATTAATAGTAATCAATTACGGGGTC**

**ATTAGTTCATAGCCCATATATGGAGTTCCGCGTTACATAACTTACGGTAAATGGCCCGCCTGGCTGACCG**

**CCCAACGACCCCCGCCCATTGACGTCAATAATGACGTATGTTCCCATAGTAACGCCAATAGGGACTTTCC**

**ATTGACGTCAATGGGTGGAGTATTTACGGTAAACTGCCCACTTGGCAGTACATCAAGTGTATCATATGCC**

**AAGTACGCCCCCTATTGACGTCAATGACGGTAAATGGCCCGCCTGGCATTATGCCCAGTACATGACCTTA**

**TGGGACTTTCCTACTTGGCAGTACATCTACGTATTAGTCATCGCTATTACCATGGTGATGCGGTTTTGGC**

**AGTACATCAATGGGCGTGGATAGCGGTTTGACTCACGGGGATTTCCAAGTCTCCACCCCATTGACGTCAA**

**TGGGAGTTTGTTTTGGCACCAAAATCAACGGGACTTTCCAAAATGTCGTAACAACTCCGCCCCATTGACG**

**CAAATGGGCGGTAGGCGTGTACGGTGGGAGGTCTATATAAGCAGAGCTCTCTGGCTAACTAGAGAACCCA**

**CTGCTTACTGGCTTATCGAAATTAATACGACTCACTATAGGGAGACCCAAGCTGGCTAGCGTTTAAACTT**

**AAGCTTGGCAATCCGGTACTGTTGGTAAATAAGCCACCGGGGTCTTCACACTCGAAGATTTCGTTGGGGA**

**CTGGCGACAGACAGCCGGCTACAACCTGGACCAAGTCCTTGAACAGGGAGGTGTGTCCAGTTTGTTTCAG**

**AATCTCGGGGTGTCCGTAACTCCGATCCAAAGGATTGTCCTGAGCGGTGAAAATGGGCTGAAGATCGACA**

**TCCATGTCATCATCCCGTATGAAGGTCTGAGCGGCGACCAAATGGGCCAGATCGAAAAAATTTTTAAGGT**

**GGTGTACCCTGTGGATGATCATCACTTTAAGGTGATCCTGCACTATGGCACACTGGTAATCGACGGGGTT**

**ACGCCGAACATGATCGACTATTTCGGACGGCCGTATGAAGGCATCGCCGTGTTCGACGGCAAAAAGATCA**

**CTGTAACAGGGACCCTGTGGAACGGCAACAAAATTATCGACGAGCGCCTGATCAACCCCGACGGCTCCCT**

**GCTGTTCCGAGTAACCATCAACGGAGTGACCGGCTGGCGGCTGTGCGAACGCATTCTGGCGGACTACAAA**

**GACCATGACGGTGATTATAAAGATCATGACATCGATTACAAGGATGACGATGACAAGTAAGGCCGCGACT**

**CTAGAGGGCCCGTTTAAACCCGCTGATCAGCCTCGACTGTGCCTTCTAGTTGCCAGCCATCTGTTGTTTG**

**CCCCTCCCCCGTGCCTTCCTTGACCCTGGAAGGTGCCACTCCCACTGTCCTTTCCTAATAAAATGAGGAA**

**ATTGCATCGCATTGTCTGAGTAGGTGTCATTCTATTCTGGGGGGTGGGGTGGGGCAGGACAGCAAGGGGG**

**AGGATTGGGAAGACAATAGCAGGCATGCTGGGGATGCGGTGGGCTCTATGGCTTCTGAGGCGGAAAGAAC**

**CAGCTGGGGCTCTAGGGGGTATCCCCACGCGCCCTGTAGCGGCGCATTAAGCGCGGCGGGTGTGGTGGTT**

**ACGCGCAGCGTGACCGCTACACTTGCCAGCGCCCTAGCGCCCGCTCCTTTCGCTTTCTTCCCTTCCTTTC**

**TCGCCACGTTCGCCGGCTTTCCCCGTCAAGCTCTAAATCGGGGGCTCCCTTTAGGGTTCCGATTTAGTGC**

**TTTACGGCACCTCGACCCCAAAAAACTTGATTAGGGTGATGGTTCACGTAGTGGGCCATCGCCCTGATAG**

**ACGGTTTTTCGCCCTTTGACGTTGGAGTCCACGTTCTTTAATAGTGGACTCTTGTTCCAAACTGGAACAA**

**CACTCAACCCTATCTCGGTCTATTCTTTTGATTTATAAGGGATTTTGCCGATTTCGGCCTATTGGTTAAA**

**AAATGAGCTGATTTAACAAAAATTTAACGCGAATTAATTCTGTGGAATGTGTGTCAGTTAGGGTGTGGAA**

**AGTCCCCAGGCTCCCCAGCAGGCAGAAGTATGCAAAGCATGCATCTCAATTAGTCAGCAACCAGGTGTGG**

**AAAGTCCCCAGGCTCCCCAGCAGGCAGAAGTATGCAAAGCATGCATCTCAATTAGTCAGCAACCATAGTC**

**CCGCCCCTAACTCCGCCCATCCCGCCCCTAACTCCGCCCAGTTCCGCCCATTCTCCGCCCCATGGCTGAC**

**TAATTTTTTTTATTTATGCAGAGGCCGAGGCCGCCTCTGCCTCTGAGCTATTCCAGAAGTAGTGAGGAGG**

**CTTTTTTGGAGGCCTAGGCTTTTGCAAAAAGCTCCCGGGAGCTTGTATATCCATTTTCGGATCTGATCAA**

**GAGACAGGATGAGGATCGTTTCGCATGATTGAACAAGATGGATTGCACGCAGGTTCTCCGGCCGCTTGGG**

**TGGAGAGGCTATTCGGCTATGACTGGGCACAACAGACAATCGGCTGCTCTGATGCCGCCGTGTTCCGGCT**

**GTCAGCGCAGGGGCGCCCGGTTCTTTTTGTCAAGACCGACCTGTCCGGTGCCCTGAATGAACTGCAGGAC**

**GAGGCAGCGCGGCTATCGTGGCTGGCCACGACGGGCGTTCCTTGCGCAGCTGTGCTCGACGTTGTCACTG**

**AAGCGGGAAGGGACTGGCTGCTATTGGGCGAAGTGCCGGGGCAGGATCTCCTGTCATCTCACCTTGCTCC**

**TGCCGAGAAAGTATCCATCATGGCTGATGCAATGCGGCGGCTGCATACGCTTGATCCGGCTACCTGCCCA**

**TTCGACCACCAAGCGAAACATCGCATCGAGCGAGCACGTACTCGGATGGAAGCCGGTCTTGTCGATCAGG**

**ATGATCTGGACGAAGAGCATCAGGGGCTCGCGCCAGCCGAACTGTTCGCCAGGCTCAAGGCGCGCATGCC**

**CGACGGCGAGGATCTCGTCGTGACCCATGGCGATGCCTGCTTGCCGAATATCATGGTGGAAAATGGCCGC**

**TTTTCTGGATTCATCGACTGTGGCCGGCTGGGTGTGGCGGACCGCTATCAGGACATAGCGTTGGCTACCC**

**GTGATATTGCTGAAGAGCTTGGCGGCGAATGGGCTGACCGCTTCCTCGTGCTTTACGGTATCGCCGCTCC**

**CGATTCGCAGCGCATCGCCTTCTATCGCCTTCTTGACGAGTTCTTCTGAGCGGGACTCTGGGGTTCGAAA**

**TGACCGACCAAGCGACGCCCAACCTGCCATCACGAGATTTCGATTCCACCGCCGCCTTCTATGAAAGGTT**

**GGGCTTCGGAATCGTTTTCCGGGACGCCGGCTGGATGATCCTCCAGCGCGGGGATCTCATGCTGGAGTTC**

**TTCGCCCACCCCAACTTGTTTATTGCAGCTTATAATGGTTACAAATAAAGCAATAGCATCACAAATTTCA**

**CAAATAAAGCATTTTTTTCACTGCATTCTAGTTGTGGTTTGTCCAAACTCATCAATGTATCTTATCATGT**

**CTGTATACCGTCGACCTCTAGCTAGAGCTTGGCGTAATCATGGTCATAGCTGTTTCCTGTGTGAAATTGT**

**TATCCGCTCACAATTCCACACAACATACGAGCCGGAAGCATAAAGTGTAAAGCCTGGGGTGCCTAATGAG**

**TGAGCTAACTCACATTAATTGCGTTGCGCTCACTGCCCGCTTTCCAGTCGGGAAACCTGTCGTGCCAGCT**

**GCATTAATGAATCGGCCAACGCGCGGGGAGAGGCGGTTTGCGTATTGGGCGCTCTTCCGCTTCCTCGCTC**

**ACTGACTCGCTGCGCTCGGTCGTTCGGCTGCGGCGAGCGGTATCAGCTCACTCAAAGGCGGTAATACGGT**

**TATCCACAGAATCAGGGGATAACGCAGGAAAGAACATGTGAGCAAAAGGCCAGCAAAAGGCCAGGAACCG**

**TAAAAAGGCCGCGTTGCTGGCGTTTTTCCATAGGCTCCGCCCCCCTGACGAGCATCACAAAAATCGACGC**

**TCAAGTCAGAGGTGGCGAAACCCGACAGGACTATAAAGATACCAGGCGTTTCCCCCTGGAAGCTCCCTCG**

**TGCGCTCTCCTGTTCCGACCCTGCCGCTTACCGGATACCTGTCCGCCTTTCTCCCTTCGGGAAGCGTGGC**

**GCTTTCTCATAGCTCACGCTGTAGGTATCTCAGTTCGGTGTAGGTCGTTCGCTCCAAGCTGGGCTGTGTG**

**CACGAACCCCCCGTTCAGCCCGACCGCTGCGCCTTATCCGGTAACTATCGTCTTGAGTCCAACCCGGTAA**

**GACACGACTTATCGCCACTGGCAGCAGCCACTGGTAACAGGATTAGCAGAGCGAGGTATGTAGGCGGTGC**

**TACAGAGTTCTTGAAGTGGTGGCCTAACTACGGCTACACTAGAAGAACAGTATTTGGTATCTGCGCTCTG**

**CTGAAGCCAGTTACCTTCGGAAAAAGAGTTGGTAGCTCTTGATCCGGCAAACAAACCACCGCTGGTAGCG**

**GTGGTTTTTTTGTTTGCAAGCAGCAGATTACGCGCAGAAAAAAAGGATCTCAAGAAGATCCTTTGATCTT**

**TTCTACGGGGTCTGACGCTCAGTGGAACGAAAACTCACGTTAAGGGATTTTGGTCATGAGATTATCAAAA**

**AGGATCTTCACCTAGATCCTTTTAAATTAAAAATGAAGTTTTAAATCAATCTAAAGTATATATGAGTAAA**

**CTTGGTCTGACAGTTACCAATGCTTAATCAGTGAGGCACCTATCTCAGCGATCTGTCTATTTCGTTCATC**

**CATAGTTGCCTGACTCCCCGTCGTGTAGATAACTACGATACGGGAGGGCTTACCATCTGGCCCCAGTGCT**

**GCAATGATACCGCGAGACCCACGCTCACCGGCTCCAGATTTATCAGCAATAAACCAGCCAGCCGGAAGGG**

**CCGAGCGCAGAAGTGGTCCTGCAACTTTATCCGCCTCCATCCAGTCTATTAATTGTTGCCGGGAAGCTAG**

**AGTAAGTAGTTCGCCAGTTAATAGTTTGCGCAACGTTGTTGCCATTGCTACAGGCATCGTGGTGTCACGC**

**TCGTCGTTTGGTATGGCTTCATTCAGCTCCGGTTCCCAACGATCAAGGCGAGTTACATGATCCCCCATGT**

**TGTGCAAAAAAGCGGTTAGCTCCTTCGGTCCTCCGATCGTTGTCAGAAGTAAGTTGGCCGCAGTGTTATC**

**ACTCATGGTTATGGCAGCACTGCATAATTCTCTTACTGTCATGCCATCCGTAAGATGCTTTTCTGTGACT**

**GGTGAGTACTCAACCAAGTCATTCTGAGAATAGTGTATGCGGCGACCGAGTTGCTCTTGCCCGGCGTCAA**

**TACGGGATAATACCGCGCCACATAGCAGAACTTTAAAAGTGCTCATCATTGGAAAACGTTCTTCGGGGCG**

**AAAACTCTCAAGGATCTTACCGCTGTTGAGATCCAGTTCGATGTAACCCACTCGTGCACCCAACTGATCT**

**TCAGCATCTTTTACTTTCACCAGCGTTTCTGGGTGAGCAAAAACAGGAAGGCAAAATGCCGCAAAAAAGG**

**GAATAAGGGCGACACGGAAATGTTGAATACTCATACTCTTCCTTTTTCAATATTATTGAAGCATTTATCA**

**GGGTTATTGTCTCATGAGCGGATACATATTTGAATGTATTTAGAAAAATAAACAAATAGGGGTTCCGCGC**

**ACATTTCCCCGAAAAGTGCCACCTGACGTC**

**>AUGnLuc3xFLAG**

***ATCTGCTTAGGGTTAGGCGTTTTGCGCTGCTTCGCGATGTACGGGCCAGATATACGCGTTGACATTGATT***

***ATTGACTAGTTATTAATAGTAATCAATTACGGGGTCATTAGTTCATAGCCCATATATGGAGTTCCGCGTT***

***ACATAACTTACGGTAAATGGCCCGCCTGGCTGACCGCCCAACGACCCCCGCCCATTGACGTCAATAATGA***

***CGTATGTTCCCATAGTAACGCCAATAGGGACTTTCCATTGACGTCAATGGGTGGAGTATTTACGGTAAAC***

***TGCCCACTTGGCAGTACATCAAGTGTATCATATGCCAAGTACGCCCCCTATTGACGTCAATGACGGTAAA***

***TGGCCCGCCTGGCATTATGCCCAGTACATGACCTTATGGGACTTTCCTACTTGGCAGTACATCTACGTAT***

***TAGTCATCGCTATTACCATGGTGATGCGGTTTTGGCAGTACATCAATGGGCGTGGATAGCGGTTTGACTC***

***ACGGGGATTTCCAAGTCTCCACCCCATTGACGTCAATGGGAGTTTGTTTTGGCACCAAAATCAACGGGAC***

***TTTCCAAAATGTCGTAACAACTCCGCCCCATTGACGCAAATGGGCGGTAGGCGTGTACGGTGGGAGGTCT***

***ATATAAGCAGAGCTCTCTGGCTAACTAGAGAACCCACTGCTTACTGGCTTATCGAAATTAATACGACTCA***

***CTATAGGGAGACCCAAGCTGGCTAGCGTTTAAACTTAAGCTTGGCAATCCGGTACTGTTGGTAAATAAGC***

***CACCATGGTCTTCACACTCGAAGATTTCGTTGGGGACTGGCGACAGACAGCCGGCTACAACCTGGACCAA***

***GTCCTTGAACAGGGAGGTGTGTCCAGTTTGTTTCAGAATCTCGGGGTGTCCGTAACTCCGATCCAAAGGA***

***TTGTCCTGAGCGGTGAAAATGGGCTGAAGATCGACATCCATGTCATCATCCCGTATGAAGGTCTGAGCGG***

***CGACCAAATGGGCCAGATCGAAAAAATTTTTAAGGTGGTGTACCCTGTGGATGATCATCACTTTAAGGTG***

***ATCCTGCACTATGGCACACTGGTAATCGACGGGGTTACGCCGAACATGATCGACTATTTCGGACGGCCGT***

***ATGAAGGCATCGCCGTGTTCGACGGCAAAAAGATCACTGTAACAGGGACCCTGTGGAACGGCAACAAAAT***

***TATCGACGAGCGCCTGATCAACCCCGACGGCTCCCTGCTGTTCCGAGTAACCATCAACGGAGTGACCGGC***

***TGGCGGCTGTGCGAACGCATTCTGGCGGACTACAAAGACCATGACGGTGATTATAAAGATCATGACATCG***

***ATTACAAGGATGACGATGACAAGAATTCTGCTTGCAAGAACTGGTTCAGTAGCTTAAGCCACTTTGTGAT***

***CCACCTTAACAGCCACGGCTTTCCGCCTGAGGTTGAAGAGCAAGCCGCCGGTACATTGCCTATGTCCTGC***

***GCACAAGAAAGCGGTATGGACCGGCACCCAGCCGCTTGTGCTTCAGCTCGCATCAACGTCTAAGGCCGCG***

***ACTCTAGAGGGCCCGTTTAAACCCGCTGATCAGCCTCGACTGTGCCTTCTAGTTGCCAGCCATCTGTTGT***

***TTGCCCCTCCCCCGTGCCTTCCTTGACCCTGGAAGGTGCCACTCCCACTGTCCTTTCCTAATAAAATGAG***

***GAAATTGCATCGCATTGTCTGAGTAGGTGTCATTCTATTCTGGGGGGTGGGGTGGGGCAGGACAGCAAGG***

***GGGAGGATTGGGAAGACAATAGCAGGCATGCTGGGGATGCGGTGGGCTCTATGGCTTCTGAGGCGGAAAG***

***AACCAGCTGGGGCTCTAGGGGGTATCCCCACGCGCCCTGTAGCGGCGCATTAAGCGCGGCGGGTGTGGTG***

***GTTACGCGCAGCGTGACCGCTACACTTGCCAGCGCCCTAGCGCCCGCTCCTTTCGCTTTCTTCCCTTCCT***

***TTCTCGCCACGTTCGCCGGCTTTCCCCGTCAAGCTCTAAATCGGGGGCTCCCTTTAGGGTTCCGATTTAG***

***TGCTTTACGGCACCTCGACCCCAAAAAACTTGATTAGGGTGATGGTTCACGTAGTGGGCCATCGCCCTGA***

***TAGACGGTTTTTCGCCCTTTGACGTTGGAGTCCACGTTCTTTAATAGTGGACTCTTGTTCCAAACTGGAA***

***CAACACTCAACCCTATCTCGGTCTATTCTTTTGATTTATAAGGGATTTTGCCGATTTCGGCCTATTGGTT***

***AAAAAATGAGCTGATTTAACAAAAATTTAACGCGAATTAATTCTGTGGAATGTGTGTCAGTTAGGGTGTG***

***GAAAGTCCCCAGGCTCCCCAGCAGGCAGAAGTATGCAAAGCATGCATCTCAATTAGTCAGCAACCAGGTG***

***TGGAAAGTCCCCAGGCTCCCCAGCAGGCAGAAGTATGCAAAGCATGCATCTCAATTAGTCAGCAACCATA***

***GTCCCGCCCCTAACTCCGCCCATCCCGCCCCTAACTCCGCCCAGTTCCGCCCATTCTCCGCCCCATGGCT***

***GACTAATTTTTTTTATTTATGCAGAGGCCGAGGCCGCCTCTGCCTCTGAGCTATTCCAGAAGTAGTGAGG***

***AGGCTTTTTTGGAGGCCTAGGCTTTTGCAAAAAGCTCCCGGGAGCTTGTATATCCATTTTCGGATCTGAT***

***CAAGAGACAGGATGAGGATCGTTTCGCATGATTGAACAAGATGGATTGCACGCAGGTTCTCCGGCCGCTT***

***GGGTGGAGAGGCTATTCGGCTATGACTGGGCACAACAGACAATCGGCTGCTCTGATGCCGCCGTGTTCCG***

***GCTGTCAGCGCAGGGGCGCCCGGTTCTTTTTGTCAAGACCGACCTGTCCGGTGCCCTGAATGAACTGCAG***

***GACGAGGCAGCGCGGCTATCGTGGCTGGCCACGACGGGCGTTCCTTGCGCAGCTGTGCTCGACGTTGTCA***

***CTGAAGCGGGAAGGGACTGGCTGCTATTGGGCGAAGTGCCGGGGCAGGATCTCCTGTCATCTCACCTTGC***

***TCCTGCCGAGAAAGTATCCATCATGGCTGATGCAATGCGGCGGCTGCATACGCTTGATCCGGCTACCTGC***

***CCATTCGACCACCAAGCGAAACATCGCATCGAGCGAGCACGTACTCGGATGGAAGCCGGTCTTGTCGATC***

***AGGATGATCTGGACGAAGAGCATCAGGGGCTCGCGCCAGCCGAACTGTTCGCCAGGCTCAAGGCGCGCAT***

***GCCCGACGGCGAGGATCTCGTCGTGACCCATGGCGATGCCTGCTTGCCGAATATCATGGTGGAAAATGGC***

***CGCTTTTCTGGATTCATCGACTGTGGCCGGCTGGGTGTGGCGGACCGCTATCAGGACATAGCGTTGGCTA***

***CCCGTGATATTGCTGAAGAGCTTGGCGGCGAATGGGCTGACCGCTTCCTCGTGCTTTACGGTATCGCCGC***

***TCCCGATTCGCAGCGCATCGCCTTCTATCGCCTTCTTGACGAGTTCTTCTGAGCGGGACTCTGGGGTTCG***

***AAATGACCGACCAAGCGACGCCCAACCTGCCATCACGAGATTTCGATTCCACCGCCGCCTTCTATGAAAG***

***GTTGGGCTTCGGAATCGTTTTCCGGGACGCCGGCTGGATGATCCTCCAGCGCGGGGATCTCATGCTGGAG***

***TTCTTCGCCCACCCCAACTTGTTTATTGCAGCTTATAATGGTTACAAATAAAGCAATAGCATCACAAATT***

***TCACAAATAAAGCATTTTTTTCACTGCATTCTAGTTGTGGTTTGTCCAAACTCATCAATGTATCTTATCA***

***TGTCTGTATACCGTCGACCTCTAGCTAGAGCTTGGCGTAATCATGGTCATAGCTGTTTCCTGTGTGAAAT***

***TGTTATCCGCTCACAATTCCACACAACATACGAGCCGGAAGCATAAAGTGTAAAGCCTGGGGTGCCTAAT***

***GAGTGAGCTAACTCACATTAATTGCGTTGCGCTCACTGCCCGCTTTCCAGTCGGGAAACCTGTCGTGCCA***

***GCTGCATTAATGAATCGGCCAACGCGCGGGGAGAGGCGGTTTGCGTATTGGGCGCTCTTCCGCTTCCTCG***

***CTCACTGACTCGCTGCGCTCGGTCGTTCGGCTGCGGCGAGCGGTATCAGCTCACTCAAAGGCGGTAATAC***

***GGTTATCCACAGAATCAGGGGATAACGCAGGAAAGAACATGTGAGCAAAAGGCCAGCAAAAGGCCAGGAA***

***CCGTAAAAAGGCCGCGTTGCTGGCGTTTTTCCATAGGCTCCGCCCCCCTGACGAGCATCACAAAAATCGA***

***CGCTCAAGTCAGAGGTGGCGAAACCCGACAGGACTATAAAGATACCAGGCGTTTCCCCCTGGAAGCTCCC***

***TCGTGCGCTCTCCTGTTCCGACCCTGCCGCTTACCGGATACCTGTCCGCCTTTCTCCCTTCGGGAAGCGT***

***GGCGCTTTCTCATAGCTCACGCTGTAGGTATCTCAGTTCGGTGTAGGTCGTTCGCTCCAAGCTGGGCTGT***

***GTGCACGAACCCCCCGTTCAGCCCGACCGCTGCGCCTTATCCGGTAACTATCGTCTTGAGTCCAACCCGG***

***TAAGACACGACTTATCGCCACTGGCAGCAGCCACTGGTAACAGGATTAGCAGAGCGAGGTATGTAGGCGG***

***TGCTACAGAGTTCTTGAAGTGGTGGCCTAACTACGGCTACACTAGAAGAACAGTATTTGGTATCTGCGCT***

***CTGCTGAAGCCAGTTACCTTCGGAAAAAGAGTTGGTAGCTCTTGATCCGGCAAACAAACCACCGCTGGTA***

***GCGGTGGTTTTTTTGTTTGCAAGCAGCAGATTACGCGCAGAAAAAAAGGATCTCAAGAAGATCCTTTGAT***

***CTTTTCTACGGGGTCTGACGCTCAGTGGAACGAAAACTCACGTTAAGGGATTTTGGTCATGAGATTATCA***

***AAAAGGATCTTCACCTAGATCCTTTTAAATTAAAAATGAAGTTTTAAATCAATCTAAAGTATATATGAGT***

***AAACTTGGTCTGACAGTTACCAATGCTTAATCAGTGAGGCACCTATCTCAGCGATCTGTCTATTTCGTTC***

***ATCCATAGTTGCCTGACTCCCCGTCGTGTAGATAACTACGATACGGGAGGGCTTACCATCTGGCCCCAGT***

***GCTGCAATGATACCGCGAGACCCACGCTCACCGGCTCCAGATTTATCAGCAATAAACCAGCCAGCCGGAA***

***GGGCCGAGCGCAGAAGTGGTCCTGCAACTTTATCCGCCTCCATCCAGTCTATTAATTGTTGCCGGGAAGC***

***TAGAGTAAGTAGTTCGCCAGTTAATAGTTTGCGCAACGTTGTTGCCATTGCTACAGGCATCGTGGTGTCA***

***CGCTCGTCGTTTGGTATGGCTTCATTCAGCTCCGGTTCCCAACGATCAAGGCGAGTTACATGATCCCCCA***

***TGTTGTGCAAAAAAGCGGTTAGCTCCTTCGGTCCTCCGATCGTTGTCAGAAGTAAGTTGGCCGCAGTGTT***

***ATCACTCATGGTTATGGCAGCACTGCATAATTCTCTTACTGTCATGCCATCCGTAAGATGCTTTTCTGTG***

***ACTGGTGAGTACTCAACCAAGTCATTCTGAGAATAGTGTATGCGGCGACCGAGTTGCTCTTGCCCGGCGT***

***CAATACGGGATAATACCGCGCCACATAGCAGAACTTTAAAAGTGCTCATCATTGGAAAACGTTCTTCGGG***

***GCGAAAACTCTCAAGGATCTTACCGCTGTTGAGATCCAGTTCGATGTAACCCACTCGTGCACCCAACTGA***

***TCTTCAGCATCTTTTACTTTCACCAGCGTTTCTGGGTGAGCAAAAACAGGAAGGCAAAATGCCGCAAAAA***

***AGGGAATAAGGGCGACACGGAAATGTTGAATACTCATACTCTTCCTTTTTCAATATTATTGAAGCATTTA***

***TCAGGGTTATTGTCTCATGAGCGGATACATATTTGAATGTATTTAGAAAAATAAACAAATAGGGGTTCCG***

***CGCACATTTCCCCGAAAAGTGCCACCTGACGTCGACGGATCGGGAGATCTCCCGATCCCCTATGGTGCAC***

***TCTCAGTACAATCTGCTCTGATGCCGCATAGTTAAGCCAGTATCTGCTCCCTGCTTGTGTGTTGGAGGTC***

***GCTGAGTAGTGCGCGAGCAAAATTTAAGCTACAACAAGGCAAGGCTTGACCGACAATTGCATGAAGA***

**SUPPLEMENTARY FIGURE**

**Fig. S1. (A) Poly-(Glu-Gly) is localized in the nucleus but does not induce apoptosis.** FLAG staining (red) of the AUG-nLuc-3xFLAG construct transfected into COS-7 cells revealed widespread nuclear and cytoplasmic distribution. In contrast, the FLAG signal shifted to the nucleus and colocalized with DAPI, a nuclear counterstain (blue), when 30 AGAGGG repeats were inserted upstream of GGG-nLuc-3xFLAG in RF2 (Poly-Glu-Gly). Poly-(Glu-Gly) showed a tendency to form inclusions. NTC = no transfection control. Scale bar = 10 μm. **(B)** Percentage of TUNEL-positive cells. Error bars represent mean ± SD.

**
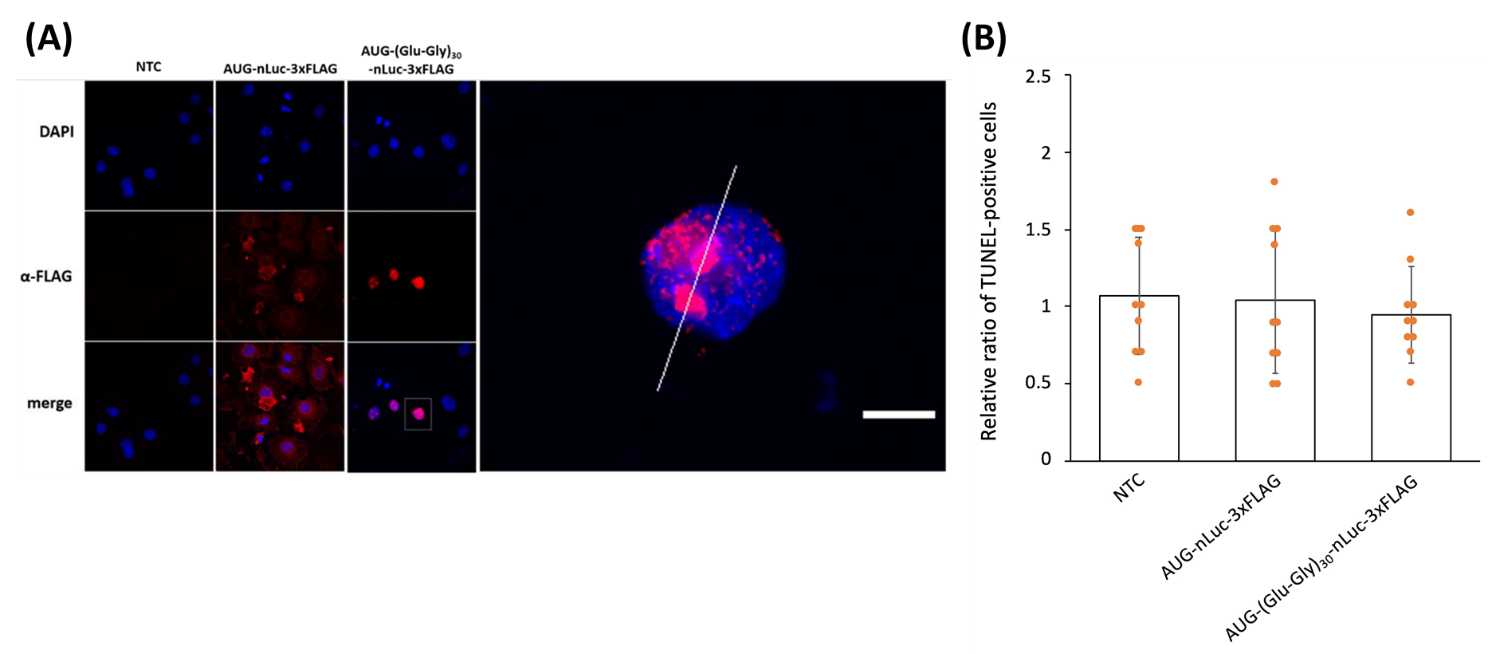
**

**SUPPLEMENTARY DISCUSSION**

Notably, the (AGAGGG)_n_ repeat within the *TAF1* SVA retrotransposon insertion was found to be translated in only two reading frames based on our reporter assays similar to the CGG repeat expansion in the *FMR1* gene causing FXTAS.^1,2^ No signal was detected in the reading frame corresponding to Poly-(Gly-Arg) or Poly-GR. Poly-GR aggregates have been shown to accumulate in the neurons of *C9ORF72* repeat expansion carriers^3,4^ and cause neurotoxicity by impairing protein translation,^5^ altering stress granule dynamics,^5^ and compromising mitochondrial function.^6,7^ Consistent with our findings, a past investigation observed limited expression of Poly-GR in transfected cell lines primarily due to inefficient translation initiation driven by an AGG start codon in the *C9ORF72* GGGGCC repeat context.^8^ While we did not detect a signal in the Poly-GR frame in our assays, it is possible that this DPR may be seen in the aged neurons of XDP patients due to a collapse of protein homeostasis.^9^

Similarly, Poly-GE expression did not appear to cause apoptosis in COS-7 cells 24-hours after transfection. This observation was also observed for Poly-GR, which followed a temporal transition from a diffuse state 24 hours post-transfection to robust aggregates 48 hours post-transfection.^5^ Thus, Poly-GE may take longer periods to aggregate and cause toxicity in the selectively vulnerable neuronal populations. Further studies utilizing patient-derived post-mortem brains are needed to confirm this possibility.

**SUPPLEMENTARY REFERENCES**

1. Todd PK, Oh SY, Krans A, et al. Article CGG Repeat-Associated Translation Mediates Neurodegeneration in Fragile X Tremor Ataxia Syndrome. *Neuron*. 2013;78(3):440-455. doi:10.1016/j.neuron.2013.03.026

2. Kearse MG, Green KM, Krans A, et al. CGG Repeat-Associated Non-AUG Translation Utilizes a Cap-Dependent Scanning Mechanism of Initiation to Produce Toxic Proteins Short Article CGG Repeat-Associated Non-AUG Translation Utilizes a Cap-Dependent Scanning Mechanism of Initiation to Produce Toxi. *Mol Cell*. 2016;62(2):314-322. doi:10.1016/j.molcel.2016.02.034

3. Mori K, Weng S ming, Arzberger T, et al. The C9orf72 GGGGCC Repeat Is Translated into Aggregating Dipeptide-Repeat Proteins in FTLD/ALS. *Science (80- )*. 2013;339(6125):1335-1338. doi:10.1126/science.1232927

4. Ash PEA, Bieniek KF, Gendron TF, et al. Unconventional Translation of C9ORF72 GGGGCC Expansion Generates Insoluble Polypeptides Specific to c9FTD/ALS. *Neuron*. 2013;77(4):639-646. doi:10.1016/j.neuron.2013.02.004

5. Zhang YJ, Gendron TF, Ebbert MTW, et al. Poly(GR) impairs protein translation and stress granule dynamics in C9orf72-associated frontotemporal dementia and amyotrophic lateral sclerosis. *Nat Med*. 2018;24(8):1136-1142. doi:10.1038/s41591-018-0071-1

6. Lopez-Gonzalez R, Lu Y, Gendron TF, et al. Poly(GR) in C9ORF72-Related ALS/FTD Compromises Mitochondrial Function and Increases Oxidative Stress and DNA Damage in iPSC-Derived Motor Neurons. *Neuron*. 2016;92(2):383-391. doi:10.1016/j.neuron.2016.09.015

7. Choi SY, Lopez-Gonzalez R, Krishnan G, et al. C9ORF72-ALS/FTD-associated poly(GR) binds Atp5a1 and compromises mitochondrial function in vivo. *Nat Neurosci*. 2019;22(6):851-862. doi:10.1038/s41593-019-0397-0

8. Boivin M, Pfister V, Gaucherot A, et al. Reduced autophagy upon C9ORF72 loss synergizes with dipeptide repeat protein toxicity in G4C2 repeat expansion disorders. *EMBO J*. 2020;39(4):1-15. doi:10.15252/embj.2018100574

9. Victor MB, Richner M, Olsen HE, et al. Striatal neurons directly converted from Huntington’s disease patient fibroblasts recapitulate age-associated disease phenotypes. *Nat Neurosci*. Published online 2018:1-12. doi:10.1038/s41593-018-0075-7
